# Incidence of Neonatal Seizures in China Based on Electroencephalogram Monitoring in Neonatal Neurocritical Care Units

**DOI:** 10.1001/jamanetworkopen.2023.26301

**Published:** 2023-07-28

**Authors:** Kai Yan, Guoqiang Cheng, Wei Zhou, Feifan Xiao, Chongfan Zhang, Laishuan Wang, Peng Zhang, Chunmei Lu, Yanting Kong, Xinhua Wang, Yuanfeng Zhou, Weineng Lu, Juan Tang, Xiaoyan Song, Qiufen Wei, Danhua Meng, Liping Yao, Deyi Zhuang, Liuhong Qu, Qinghuo Xu, Zhaoqing Yin, Le Su, Jing Wan, Yuan Si, Kazumichi Fujioka, Michele Mussap, Shibani Kanungo, Vineet Bhandari, Weimin Huang, Xinnian Pan, Wenhao Zhou

**Affiliations:** 1 Department of Neonatology, Children’s Hospital of Fudan University, Shanghai, China; 2 Department of Neonatology, Guangzhou Women & Children’s Medical Center, Guangdong, China; 3 Department of Neurology, Children’s Hospital of Fudan University, Shanghai, China; 4 Department of Neonatology, Southern Hospital of Southern Medical University, Guangdong, China; 5 Department of Neonatology, The Maternal and Child Health Hospital of Guangxi Zhuang Autonomous Region, Guangxi Zhuang Autonomous Region, Guangxi, China; 6 Department of Neonatology, Xiamen Children’s Hospital, Fujian, China; 7 Department of Neonatology, Guangzhou Huadu District Maternal and Child Health Hospital, Guangdong, China; 8 Department of Neonatology, Dehong People’s Hospital of Yunnan Province, Yunnan, China; 9 Department of Pediatrics, Kobe University Graduate School of Medicine, Kobe, Japan; 10 Laboratory Medicine, Department of Surgical Sciences, University of Cagliari, Cittadella Universitaria S.S. 554, Monserrato, Cagliari, Italy; 11 Department of Pediatric and Adolescent Medicine, Western Michigan University Homer Stryker MD School of Medicine, Kalamazoo; 12 Section of Neonatology, Department of Pediatrics, St Christopher’s Hospital for Children, Drexel University College of Medicine, Philadelphia, Pennsylvania

## Abstract

**Importance:**

Neonatal seizures pose a significant challenge in critical care, and continuous video electroencephalography (cEEG) monitoring holds promise for early detection of seizures. However, large-scale data on the incidence of neonatal seizures and monitoring systems in China are lacking.

**Objectives:**

To determine the incidence of neonatal seizures in infants with high risk in China.

**Design, Setting, and Participants:**

A large, cross-sectional multicenter study was conducted from January 2017 to December 2018 in the neonatal intensive care units (NICUs) of 7 tertiary medical centers in China. Neonates with high risk were included, and cEEG monitoring was conducted. Data were collected between January 1, 2017, and January 31, 2020. The data were analyzed between January 2021 and January 2022.

**Main Outcomes and Measures:**

The incidence of neonatal seizures, categorized by etiology, and seizure burden.

**Results:**

A total of 20 310 neonates with high risk were included (10 495 [51.7%] male; mean [SD] postmenstrual age, 37.7 [3.7] weeks), and seizures were observed in 3423 infants (16.9%). The highest proportion of seizures was attributed to acute neonatal encephalopathy (1448 [42.3%]). The incidence of seizures decreased with postmenstrual age and birth weight, with the highest occurrence observed in neonates with postmenstrual age of less than 28 weeks (237 of 879 [27.0%]) or birth weight of less than 1.0 kg (269 of 914 [29.4%]). Preterm infants had a higher proportion of moderate and severe seizure burdens compared with full-term infants (moderate severity: 248 of 1199 [20.7%] vs 454 of 2224 [20.4%]), but no significant differences were observed in etiology. Seizure burden was highest with genetic syndromes (49 of 188 [26.1%]), central nervous system malformations (33 of 127 [26.0%]), and inborn errors of metabolism (27 of 113 [23.9%]). During hospitalization, 7.8% of neonates with seizures died (267 neonates), with 81.3% of these cases having a moderate or severe seizure burden (217 neonates). Mortality was generally higher in preterm vs full-term infants (98 of 1199 [8.2%] vs 169 of 2224 [7.6%]) and increased with the severity of seizure burden (217 of 267 neonates with moderate or severe burden [81.3%]).

**Conclusions and Relevance:**

This cross-sectional study of neonatal seizures underscores the substantial burden seizures pose to high-risk infants with brain injury in China, particularly those who are born prematurely or who have congenital conditions.

## Key Points

**Question:**

What is the incidence of neonatal seizures in China, and what are the implications for neonatal neurocritical care units based on continuous video electroencephalography monitoring?

**Findings:**

This cross-sectional study involving 20 310 high-risk neonates in China found an incidence of seizures in 16.9% of cases, with acute neonatal encephalopathy being the most common cause.

**Meaning:**

The findings of this study highlight the need to expand neonatal neurocritical care units and provide important insights into the incidence of neonatal seizures in China, emphasizing the significant burden they pose to high-risk infants.

## Teaser

This cross-sectional study examines the incidence of neonatal seizures in infants with high risk in China using continuous video electroencephalography monitoring.

## Introduction

Advancements in neonatal critical care techniques have contributed to a further reduction in global neonatal mortality rates.^1^ However, it is accompanied by the potential for severe neurological complications in a subset of treated neonates, who often develop brain injury or encephalopathy. Seizures are the most common manifestation of neonatal brain injury, with approximately 20% later evolving into epilepsy or neurobehavioral developmental disorders.^2^ The incidence of neonatal seizures varies, ranging from approximately 0.95 to 5.0 per 1000 live births.^3^ This rate is affected by factors such as gestational age, etiology, and medical center.^3^ With respect to etiology, neonates with hypoxic-ischemic encephalopathy exhibit the highest incidence of seizures.^4,5^ However, the incidence of neonatal seizures varies across different regions of the world, and large-scale data on the local incidence of neonatal seizures in China have yet to be reported.

Neonatal convulsive seizures often prove challenging to identify based solely on clinical observations, with roughly half or more being subclinical in nature.^6,7,8^ The American Clinical Neurophysiology Society (ACNS) states that continuous electroencephalography (cEEG) monitoring is the criterion standard for detecting seizures in high-risk neonates.^9^ Early detection of neonatal seizures may help prevent or reduce brain injuries and fatalities, and timely intervention could improve neurodevelopmental outcomes for nearly half of neonates with seizures by decreasing cerebral palsy, intellectual disability, and epileptic encephalopathy.^10^ Early electrophysiological evaluation is increasingly favored for newborns at high risk of brain injury, owing to the growing body of evidence supporting better seizure control and potentially improved outcomes.^11^

In 2009, the Children’s Hospital of Fudan University established the first neonatal neurocritical care unit in China.^12^ Since then, the development of neonatal neurocritical care techniques and monitoring units has gradually expanded to neonatal intensive care units (NICUs) in various provinces in China. In response to the absence of a large sample study cohort on neonatal neurocritical care in China, the China Neonatal Neuro-Critical Care Network (CNNCCN) was established in 2017. This consortium invited and enrolled voluntary members, standardized cEEG monitoring alongside other corresponding brain protection strategies in the NICUs of member institutions, and investigated the incidence of neonatal seizures in China through a large-scale, multicenter study.

## Methods

### Study Design

This large cross-sectional multicenter study was conducted in the NICUs of 7 tertiary medical centers. The study is registered on ClinicalTrials.gov (NCT02552511) and adheres to the guidelines of the Declaration of Helsinki.^13^ The Ethics Committee of the Children’s Hospital of Fudan University reviewed the study’s content. The parents or guardians of included newborns provided written informed consent. This study followed the Strengthening the Reporting of Observational Studies in Epidemiology (STROBE) reporting guideline.

### Participants and Procedures

Neonates from 7 level III and IV NICUs are included, with each unit admitting more than 2500 neonates and discharging between 2500 and 3500 neonates annually. Among these, those at high risk for brain injury are monitored with cEEG upon admission, in accordance with the recommendations of the ACNS guidelines.^14^ The standout features of these institutions include their advanced facilities, professional medical and nursing staff, and extensive clinical experience. Such scale and professionalism ensure that our study has a sufficient sample size and a broad range of case types, which are of great importance to our research objectives. The study period spanned from January 1, 2017, to December 31, 2018, during which a continuous sample of high-risk infants admitted to the hospital was collected.

### Sample Size Estimation

Sample size estimation was calculated using PASS 2008 version 8.0.3 (NCSS). The incidence of seizures in the NICU ranged from 0.95 to 5.0 per 1000 live births. We conservatively selected an incidence of 0.95 per 1000 live births for neonatal seizures. Assuming an allowable error of 1.0% and a 2-sided significance level of .05, a minimum of 3303 neonates with seizures would be needed. Consequently, each hospital required approximately 472 neonates with seizures.

### Monitoring Criteria of CNNCCN and cEEG Monitoring Standards

**Figure 1.**
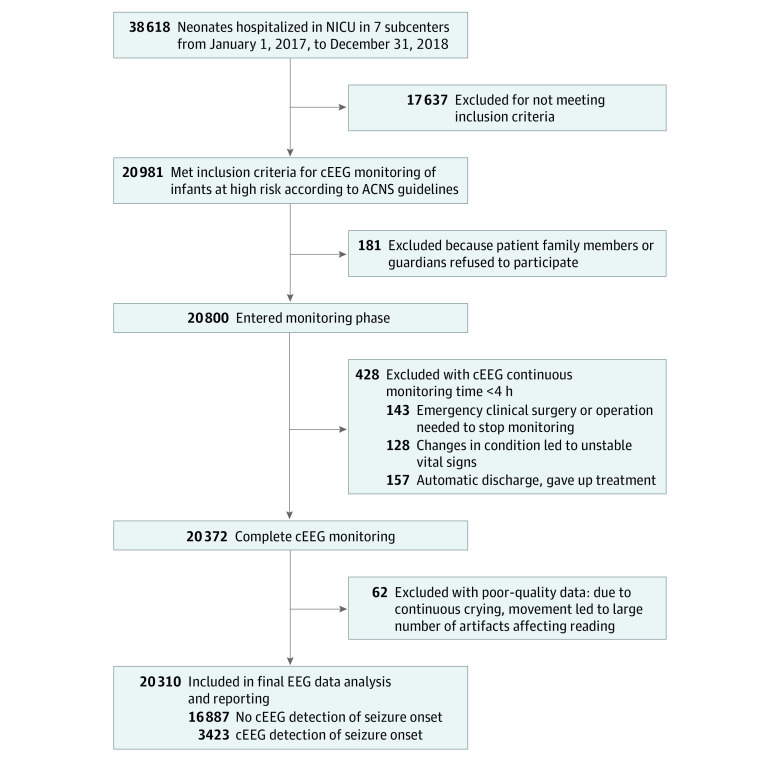
Flowchart of China Neonatal Neuro-Critical Care Network Study ACNS indicates American Clinical Neurophysiology Society; cEEG, continuous electroencephalography; NICU, neonatal intensive care unit.

Neonates at high risk for seizures, requiring cEEG monitoring per ACNS guidelines,^9^ were eligible for the study. All included newborns were no older than 28 days of age. The parents or guardians of eligible newborns were approached and provided consent. The definition and inclusion criteria for high-risk infants were referenced from the neonatal population recommended for monitoring by ACNS.^9,15^ These inclusion criteria were specifically targeted at neonates at a high risk of seizures (eTable 1 in Supplement 1). Neonates with scalp injuries, scalp infections, or scalp bleeding, which could lead to delayed EEG monitoring, were excluded from the study. Further details can be found in the study flowchart (Figure 1). Monitoring standards of cEEG can be seen in eTable 2 in Supplement 1.

### cEEG Monitoring Process

The cEEG recordings were performed using the Nicolet Monitor (Natus) video EEG system, with 32 gold-over-silver scalp cup electrodes positioned according to the International 10-20 system. Considering the small scalp area of neonates, which limits the placement of too many electrodes, and their delicate skin, which may lead to scalp injuries or pressure sore infections, we followed the recommendations of the ACNS guidelines and used 11 electrodes for monitoring (eFigure in Supplement 1). Leads FP1 and FP2 monitored the frontal pole, F3 and F4 monitored the frontal area, C3 and C4 the parietal area, T3 and T4 the temporal area, and O1 and O2 the occipital area. Three reference leads were placed: the central vertex; a heart reference lead; and a muscle reference lead.

### Etiology Clarification

The team carried out a systematic and in-depth evaluation of each possible cause of seizures in newborns, with each cause following specific diagnostic criteria (eTable 3 in Supplement 1). When evaluating acute neonatal encephalopathy (ANE), the research team carefully analyzed the relationship between other causes and ANE. If a particular cause was found to be closely related to ANE, it was considered the main cause. After ruling out all other causes, ANE was confirmed as the diagnosis. Furthermore, for infants whose causes could not be confirmed, the team made an etiological diagnosis through multidisciplinary discussion. Seizures that could not be confirmed to be caused by these causes were marked as unknown etiology.

### Baseline Data Collection

Data were collected between January 1, 2017, and January 31, 2020. Baseline information and clinical variables included: gender, postmenstrual age (PMA), birth weight, Apgar scores at 5 and 10 minutes of life, maternal diseases during pregnancy (eg, hypertension, preeclampsia, diabetes, hypothyroidism, liver dysfunction, kidney dysfunction, immune disorder, polyhydramnios/oligohydramnios [P/O], intrauterine infections), placental abruption, premature rupture of membranes, placenta previa, neonatal respiratory distress syndrome, persistent pulmonary hypertension of the newborn, multiple malformations, anemia, necrotizing enterocolitis, severe jaundice, congenital diaphragmatic hernia, congenital heart disease, retinopathy of prematurity, epilepsy syndrome, mode of delivery, duration of mechanical ventilation, length of NICU stay, NICU death, and etiology.

### Definition and Diagnosis of Seizures

Neonatal seizures are identified by at least 10 seconds (or shorter if associated with clinical changes) of paroxysmal, abnormal, and sustained ictal rhythm, which is a repetitive and evolving pattern with a minimum amplitude of 2 microvolts, as observed in the cEEG.^9,15^ Neurophysiologists will only diagnose seizures when abnormal motor events are associated with abnormal electrical activity on the EEG. To compare the association between PMA and seizure burden with prognosis, the team referred to the seizure burden grouping by the Neonatal Seizure Registry group^16^ and combined it with the postintervention status to categorize the seizure burden into 3 groups: mild, moderate, and severe. Mild refers to fewer than 7 seizures during the monitoring period and seizures that can be effectively controlled after the initial use of medication. Moderate refers to more than 7 seizures or frequent recurrences during the monitoring period, but the seizures can still be controlled after using multiple single or combined medications. Severe refers to a continuous state of seizures during the monitoring period or multiple long-lasting seizures that are difficult to control through medical treatment.

### Report of cEEG Monitoring Results

Each research center is outfitted with at least 2 fixed reporting staff members who each have more than 5 years of EEG interpretation experience. This arrangement guarantees they can alternate shifts to meet the requirement of 24-hour coverage. A chief neurophysiologist, with more than 10 years of experience, is responsible for reviewing these reports to ensure their accuracy. Before the commencement of the study, all neurophysiologists responsible for reporting underwent uniform training and education and secured EEG reporting qualifications issued by the China Anti-Epilepsy Association. Additionally, the study established regular review and quality control mechanisms, which included the evaluation of randomly selected cEEG reports by an independent review team, and regular consistency checks.

### Statistical Analysis

The data were analyzed between January 2021 and January 2022. Data analysis was conducted using Stata version 13.0 (StataCorp). Clinical variables are presented as number and percentages, with mean and SD for continuous variables or median with ranges for ordinal variables. Group comparisons of quantitative data were achieved by 2 independent sample *t* tests. The between-group comparison of qualitative data was done using the χ^2^ test. *P* < .05 was considered statistically significant, and all tests were 2-tailed.

## Results

**Figure 2.**
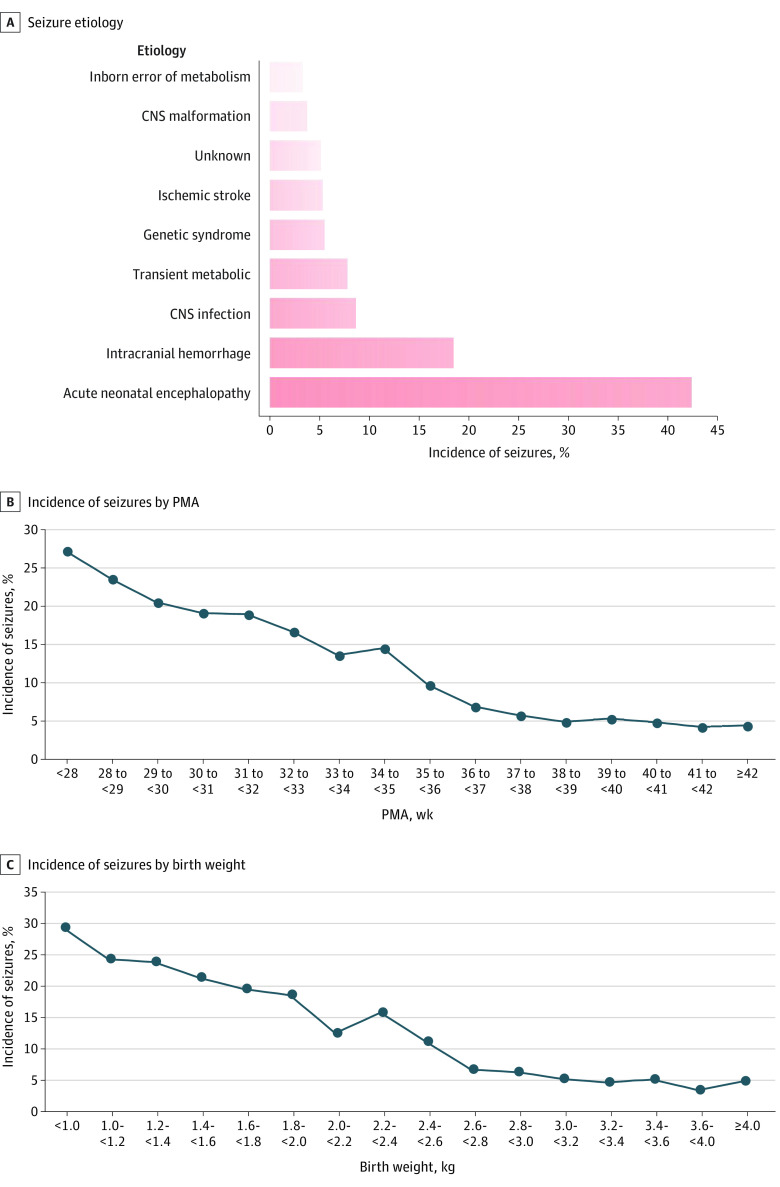
Incidence and Etiology of Neonatal Seizures in China CNS indicates central nervous system; PMA, postmenstrual age.

Overall, 20 310 neonates were recruited (10 495 [51.7%] male; mean [SD] postmenstrual age, 37.7 [3.7] weeks), and their full demographic characteristics are summarized in the Table. Figure 1 presents a flowchart detailing the operational model and recruitment process of CNNCCN as well as the number of neonates who completed EEG monitoring and those identified with seizures. Figure 2A displays the seizure etiologies for neonates undergoing cEEG monitoring. Among the 20 310 high-risk newborns, seizures occurred in 16.9% (3423). ANE accounted for 42.3% (1448), intracranial hemorrhage for 18.4% (630), central nervous system (CNS) infection for 8.6% (294), transient metabolic disorders for 7.8% (267), genetic syndromes for 5.5% (188), ischemic strokes for 5.3% (181), unknown etiology for 5.1% (175), CNS malformations for 3.7% (127), and inborn errors of metabolism for 3.3% (113).

**Table.** General and Clinical Demographics

Characteristic	Neonates, No. (%)			*P* value
	Total	Neonates with seizures	Neonates without seizures	
Total population	20 310 (100)	3423 (16.9)	16 887 (83.1)	NA
Sex				
Male	10 495 (51.7)	1754 (51.3)	8741 (51.8)	.58
Female	9815 (48.3)	1669 (48.7)	8146 (48.2)	
PMA, mean (SD), wk				
Total population	37.7 (3.72)	37.5 (4.02)	38.1 (3.89)	<.001
Preterm	35.4 (4.13)	34.5 (4.29)	35.7 (4.05)	<.001
Full-term	38.7 (3.23)	38.3 (3.57)	39.0 (2.81)	<.001
PMA, median (IQR), wk				
Total population	37.9 (35.4-40.4)	37.9 (35.2-40.6)	38.5 (35.9-41.1)	<.001
Preterm	35.0 (32.1-36.6)	33.8 (31.7-34.5)	35.4 (33.5-35.9)	<.001
Full-term	39.1 (36.9-41.0)	38.3 (35.9-40.7)	39.3 (38.1-41.4)	<.001
Birth weight, mean (SD), g				
Total population	3150 (647)	2870 (575)	3320 (736)	<.001
Preterm	2530 (893)	2340 (905)	2580 (883)	<.001
Full-term	3310 (536)	2990 (464)	3430 (432)	<.001
Birth weight, median (IQR), g				
Total population	3170 (2732-3606)	2900 (2515-3291)	3340 (2842-3836)	<.001
Preterm	2470 (1834-2640)	2340 (1732-2554)	2510 (1905-2697)	<.001
Full-term	3330 (2968-3692)	3020 (2707-3333)	3430 (3139-3723)	<.001
Apgar 5 min score, median (IQR)	9 (4-10)	5 (2-10)	9 (6-10)	<.001
Apgar 10 min score, median (IQR)	9 (5-10)	5 (4-10)	9 (8-10)	<.001
Maternal complications during pregnancy				
Hypertension	746 (3.7)	286 (8.4)	460 (2.7)	<.001
Preeclampsia	648 (3.2)	277 (8.1)	371 (2.2)	<.001
Diabetes	407 (2)	82 (2.4)	325 (1.9)	.07
Hypothyroidism	45 (0.2)	12 (0.4)	33 (0.2)	.08
Intrauterine infections	90 (0.4)	44 (1.3)	46 (0.3)	<.001
P/O	82 (0.4)	19 (0.6)	63 (0.4)	.13
Liver dysfunction	126 (0.6)	29 (0.8)	97 (0.6)	.06
Kidney dysfunction	133 (0.7)	30 (0.9)	103 (0.6)	.08
Immune disorder	45 (0.2)	12 (0.4)	33 (0.2)	.08
Placental abruption	71 (0.3)	25 (0.7)	46 (0.3)	<.001
PROM	233 (1.1)	94 (2.7)	139 (0.8)	<.001
Placenta previa	35 (0.2)	12 (0.4)	23 (0.1)	<.001
Cesarean delivery	3394 (16.7)	929 (27.2)	2465 (14.6)	.006
NRDS	4822 (23.7)	1191 (34.8)	3631 (21.5)	<.001
PPHN	89 (0.4)	21 (0.6)	68 (0.4)	.09
MM	369 (1.8)	99 (2.9)	270 (1.6)	<.001
Anemia	1909 (9.4)	507 (14.8)	1402 (8.3)	<.001
NEC	417 (2.1)	96 (2.8)	321 (1.9)	.001
Severe jaundice	244 (1.2)	58 (1.7)	186 (1.1)	.004
CDH	44 (0.2)	10 (0.3)	34 (0.2)	.30
CHD	118 (0.6)	34 (1)	84 (0.5)	.001
ROP	2245 (11.1)	387 (11.3)	1858 (11)	.61
Epilepsy syndrome	44 (0.2)	27 (0.8)	17 (0.1)	<.001
Duration of MV, mean (SD), d	7.4 (4.7)	11.0 (5.1)	6.4 (4.0)	<.001
Duration of hospitalization, mean (SD), d	25.6 (12.58)	26.5 (12.95)	24.6 (11.44)	<.001
Death during hospitalization	443 (2.2)	442 (12.9)	946 (5.6)	<.001

Abbreviations: CDH, congenital diaphragmatic hernia; CHD, congenital heart disease; MM, multiple malformations; MV, mechanical ventilation; NA, not applicable; NEC, necrotizing enterocolitis; NRDS, neonatal respiratory distress syndrome; PMA, postmenstrual age; P/O, polyhydramnios/oligohydramnios; PPHN, persistent pulmonary hypertension of the newborn; PROM, premature rupture of membranes; ROP, retinopathy of prematurity.

The incidence of neonatal seizures decreased with PMA and birth weight (Figure 2B). The highest incidence of postnatal seizures occurred in neonates with PMA of less than 28 weeks or birth weight of less than 1.0 kg, at 27.0% (237 of 879) and 29.4% (269 of 914), respectively. As gestational age and birth weight gradually increased, the incidence of neonatal seizures exhibited a slow decreasing trend. When PMA reached 36 weeks or greater or birth weight reached 2.6 kg or greater, the incidence of neonatal seizures stabilized, ranging between 3.6% and 6.9%.

**Figure 3.**
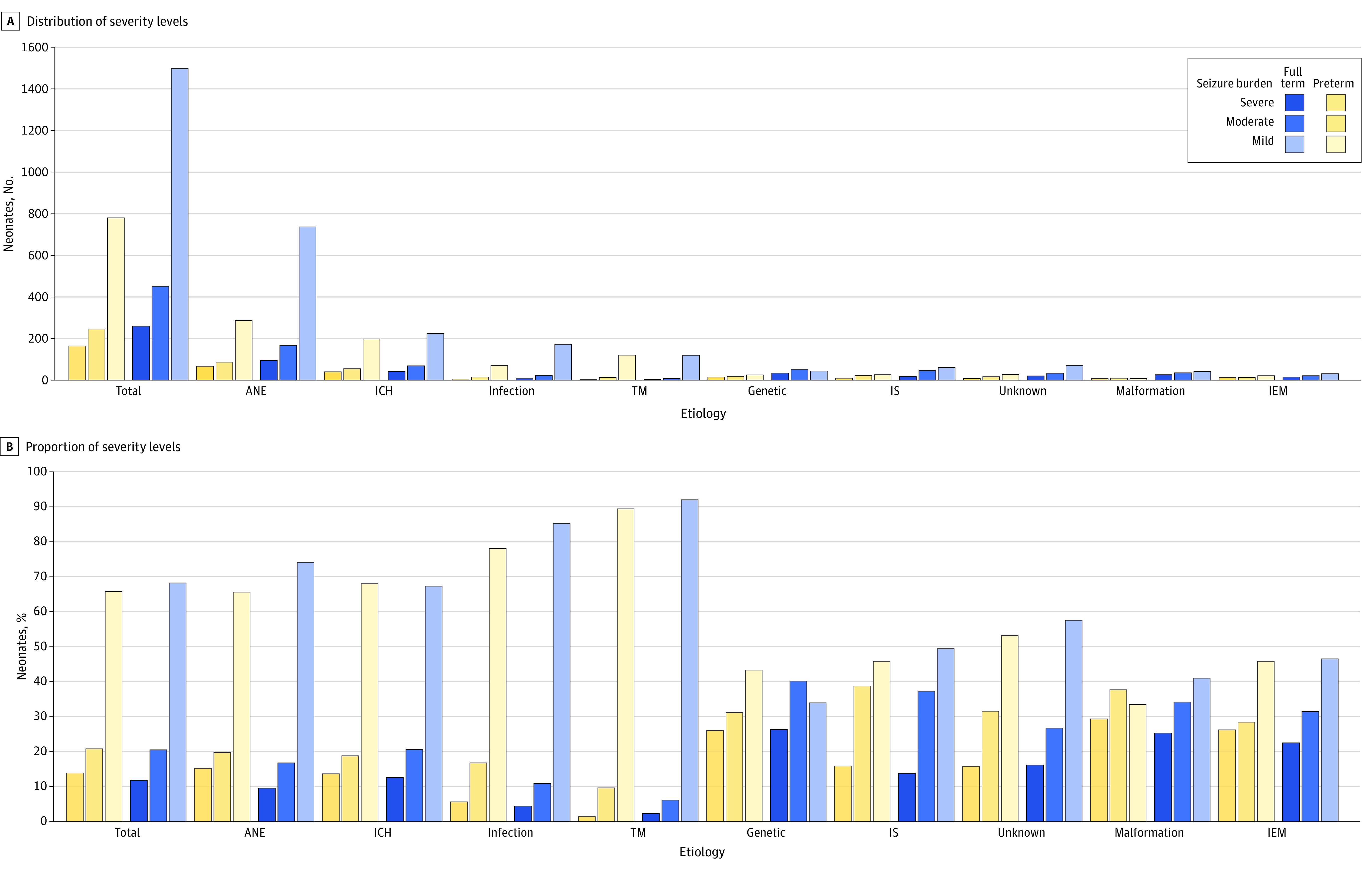
Seizure Burden in Neonates With Different Postmenstrual Ages and Etiologies Figure 3 shows the distribution of seizure burden in newborns under different etiologies and postmenstrual age. ANE indicates acute neonatal encephalopathy; ICH, intracranial hemorrhage; IEM, inborn error of metabolism; IS, ischemic stroke; TM, transient metabolic disorder.

Judging purely from the data, preterm infants had a slightly higher proportion of moderate and severe seizure burden compared with full-term infants, with moderate and severe cases accounting for 20.7% (248 of 1199) and 13.8% (165 of 1199), respectively (Figure 3; eTable 4 in Supplement 1). Conversely, full-term newborns had a lower proportion of moderate and severe seizure burden, with 20.4% (454 of 2224) for moderate and 11.7% (261 of 2224) for severe. However, compared with full-term infants, apart from the etiology of ANE, no significant differences were observed in the overall comparisons and comparisons between the 2 groups with other etiologies. In the comparison of seizure burden by etiology, genetic syndromes (49 of 188 [26.1%]), CNS malformations (33 of 127 [26.0%]), inborn errors of metabolism (27 of 113 [23.9%]), and unknown etiology (28 of 175 [16.0%]) had a more severe burden than ANE (162 of 1448 [11.2%]), intracranial hemorrhage (82 of 630 [13.0%]), CNS infection (14 of 294 [4.8%]), and transient metabolism (5 of 267 [1.9%]). Among these, transient metabolic issues had the lowest seizure burden, while genetic syndromes, CNS malformations, and inborn errors of metabolism had the highest burden.

**Figure 4.**
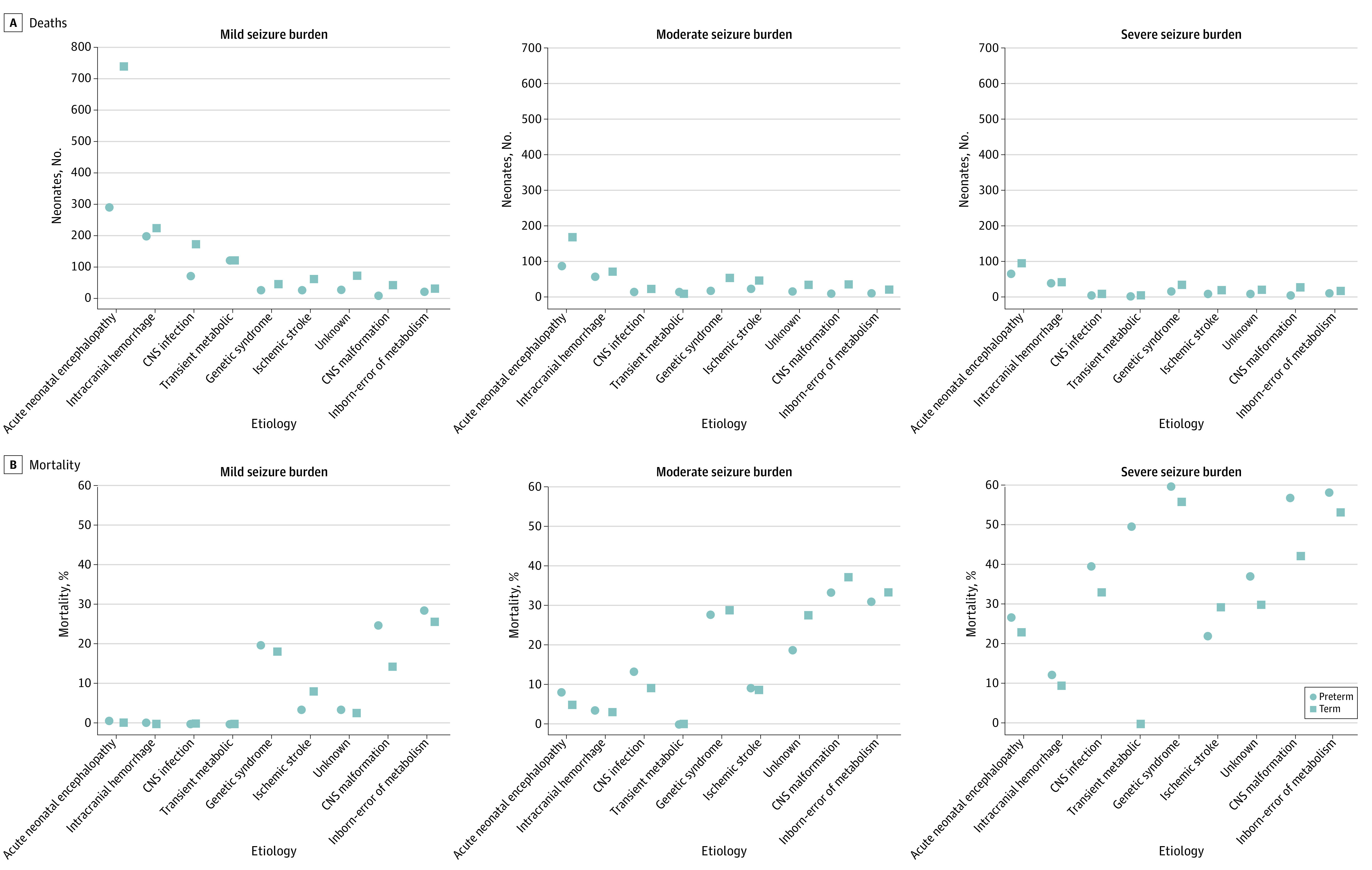
Postmenstrual Age, Seizure Burden, and Neonatal Deaths and Mortality CNS indicates central nervous system.

A total of 7.8% of neonates with seizures (267) died during hospitalization (Figure 4). Among the deceased newborns, 217 (81.3%) experienced a moderate or severe seizure burden. In the NICU, 98 preterm infants with seizures (8.2%) died, with 79 (80.6%) having a moderate or severe seizure burden. On the other hand, in the full-term group, 169 (7.6%) died during their NICU stay, and 138 (81.7%) faced a moderate or severe seizure burden. When comparing different etiologies, the mortality rate was generally higher in preterm births than in full-term births. The lowest mortality rate was associated with transient metabolic disorders, followed by CNS infection and intracranial hemorrhage. In contrast, the 3 highest mortality causes were inborn errors of metabolism, CNS malformations, and genetic syndromes.

Across all etiologies, the number of neonates with seizures decreased as the seizure burden increased, but the mortality rate rose with the severity of the seizure burden (Figure 4). This pattern was evident in both preterm and full-term infants. Although the number of deaths among preterm infants with moderate-to-severe seizure burden was lower than in full-term infants, the mortality rate was higher (98 of 1199 [8.2%] vs 169 of 2224 [7.6%]). The lower the PMA and the more severe the seizure burden, the higher the mortality rate during hospitalization. Conversely, the greater the PMA and the milder the seizure burden, the higher the survival rate during hospitalization.

## Discussion

Seizures are the most prevalent neurological symptom in newborns with acute brain injury.^17^ cEEG monitoring serves as the criterion standard for seizure detection.^18,19^ Over the past decade, as cEEG has been increasingly adopted for neurocritical monitoring in China,^20^ a growing number of third-level NICUs have provided newborns with the opportunity to receive brain protection upon admission. In 2011 and 2013,^9,15^ the ACNS successively released guidelines and consensus on newborn brain monitoring, providing a harmonized reference and management standard for newborn brain monitoring worldwide. As a result, the Children’s Hospital of Fudan University spearheaded the formation of the CNNCCN, which established the first neonatal neurocritical care unit in China.^21^ Encompassing more than 20 000 cases of newborn seizures, this study represents the most extensive multicenter observational investigation in China, boasting the largest sample size reported to date of which we are aware. The present study unveils the cross-sectional data of newborn seizures collected over a 2-year span.

In this study, seizures were detected in 3423 high-risk infants (16.9%), which aligns with international data.^5,22,23^ The incidence of neonatal seizures varies among studies due to factors such as sample size, location, and the level of research institutions involved.^24,25,26,27^ The global incidence of neonatal seizures is not uniform, with higher rates observed in low-resource countries than in high-resource countries and in low-income countries compared with high-income countries.^28,29^ The incidence of seizures in high-risk infants in our study was higher than in the study by Sheth et al^30^ (16.9% vs 8.6%), which may suggest a gap in the level of prevention and management of neonatal seizures in China compared with higher-resource countries. Moreover, our study found that the incidence of seizures decreased with increasing gestational age or birth weight, in line with findings from other teams.^31,32^

Studies focusing on newborns of less than 28 weeks of gestation are very limited.^33^ To our knowledge, our research represents the largest cross-sectional study to date in China concerning extremely preterm infants. Past research indicates that the incidence of seizures in preterm infants in the NICU is 2 to 10 times higher than in full-term infants.^27,34^ This might be higher than our research findings. The discrepancy could be related to sample size, as we have data for each gestational age group, with the rates for each group relatively stable. Furthermore, we found that the mortality rate was higher for preterm infants with seizures, with the rate increasing as gestational age decreased.^35^ Extremely preterm infants face a 20% to 50% risk of experiencing seizures,^36,37,38,39,40,41,42^ and the incidence of cognitive and motor disorders also significantly increases.^43^ These findings underscore the importance of early identification and management of neonatal seizures in preterm infants, given the heightened risk of adverse outcomes for extremely preterm infants. This indicates that preterm infants remain a high-risk group for brain injury in the NICU, and that brain protection strategies should be implemented during the perinatal period to improve postnatal neurodevelopmental outcomes in this population.

Perinatal environmental factors still remain the most common causes of seizures in high-risk infants.^5,22,23^ ANE accounted for 42.3%, followed by intracranial hemorrhage, CNS infections, transient metabolic disorders, and ischemic strokes. These factors together accounted for 82.4%. This underscores that perinatal scientific treatment of pregnant individuals in China remains very important. Preventing infections during pregnancy and preventing the occurrence of acute and chronic fetal asphyxia can reduce the occurrence of most neonatal seizures. With advances in gene testing and neuroimaging technology, previously elusive cases, such as genetic syndromes, CNS malformations, and congenital metabolic problems, can now be precisely diagnosed early in life. This highlights the necessity of establishing a genetic monitoring and consultation system within domestic NICUs, which can also change the outcomes for families by providing advance knowledge of their neonates’ health. However, the precise etiology of 5.1% of cases remained unknown; we still have a long way to go to clarify the whole etiological spectrum of neonatal seizures.

The etiology of seizures is closely linked with the burden and mortality rate of critically ill newborns. Although genetic syndromes, CNS malformations, and inborn errors of metabolism accounted for merely 12.5% of all seizure cases, these infants experienced severe seizure burdens and a high rate of early death. Critically ill infants with these 3 major etiologies often face severe metabolic disorders, uncontrolled seizures, progressively worsening brain damage, or death during treatment, with a poor long-term prognosis.^44^ Therefore, we emphasize the need for heightened clinical vigilance for these types of patients. Prompt activation of genetic or other auxiliary tests should be initiated as soon as suspicion arises, aiming to secure time for intervention and treatment to improve prognosis.

### Limitations

This study has limitations. First, we lacked long-term outcome data and control data for high-risk infants not monitored with cEEG due to early funding constraints. We aim to create a large cEEG monitoring cohort for comparison. Second, we may have missed seizure cases without clinical symptoms by stopping monitoring after 4 hours. Despite ACNS recommendations of at least 24 hours of monitoring, implementing such continuous, large-scale monitoring was challenging. Future research might investigate the optimal cEEG monitoring duration. Furthermore, variability in cEEG analysis and reporting levels was expected despite our training measures, emphasizing the importance of experienced neurophysiologists and adherence to ACNS guidelines.^45^ Future studies could potentially involve a larger neonatal population and long-term follow-up.

## Conclusion

This cross-sectional study highlights the considerable burden that neonatal seizures pose to high-risk infants in China, especially those born preterm or with congenital conditions. Improvements in prenatal and neurocritical care, optimization of treatments for medically refractory seizures, and expanded access to early diagnosis and coordinated care for complex conditions could help address excess morbidity and mortality.

## Notes

Supplement 1. **eFigure.** Process Diagram of the Multicenter Study **eTable 1.** Inclusion Criteria for Neonatal Seizure Screening Population **eTable 2.** Monitoring Standards of cEEG for Neonates in Neonatal Neurocritical Care Units in China **eTable 3.** Diagnostic Criteria for the Etiology of Neonates With Seizures **eTable 4.** Seizure Burden in Neonates Under Different PMA and Etiologies

Supplement 2.
Nonauthor Collaborators

Supplement 3.
Data Sharing Statement
