# Supplementary material for: Incidence of Neonatal Seizures in China Based on Electroencephalogram Monitoring in Neonatal Neurocritical Care Units
Source: JAMA Netw Open. 2023 Jul 28;6(7):e2326301. doi: 10.1001/jamanetworkopen.2023.26301 (PMC10383014; doi:10.1001/jamanetworkopen.2023.26301)
Supplement: Supplement 1. — eFigure. Process Diagram of the Multicenter Study eTable 1. Inclusion Criteria for Neonatal Seizure Screening Population eTable 2. Monitoring Standards of cEEG for Neonates in Neonatal Neurocritical Care Units in China eTable 3. Diagnostic Criteria for the Etiology of Neonates With Seizures eTable 4. Seizure Burden in Neonates Under Different PMA and Etiologies [file jamanetwopen-e2326301-s001.pdf]

Supplemental Online Content

Yan K, Cheng G, Zhou W, et al; China Neonatal Neuro-Critical Care Network group. Incidence of neonatal seizures in China based on electroencephalogram monitoring in neonatal neurocritical care units. *JAMA Netw Open.* 2023;6(7):e2326301. doi:10.1001/jamanetworkopen.2023.26301

eFigure. Process Diagram of the Multicenter Study

eTable 1. Inclusion Criteria for Neonatal Seizure Screening Population

eTable 2. Monitoring Standards of cEEG for Neonates in Neonatal Neurocritical Care Units in China

eTable 3. Diagnostic Criteria for the Etiology of Neonates With Seizures

eTable 4. Seizure Burden in Neonates Under Different PMA and Etiologies

This supplemental material has been provided by the authors to give readers additional information about their work.

© 2023 Yan K et al. *JAMA Network Open.*

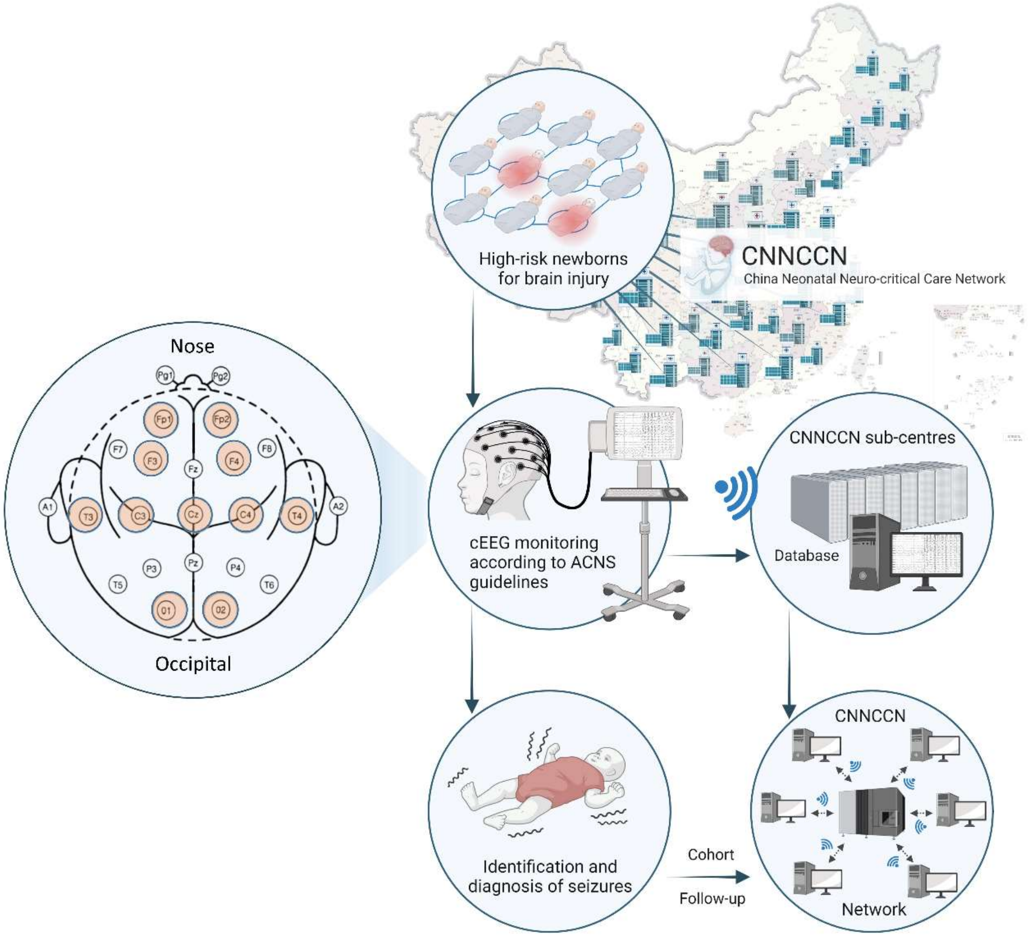

eFigure. Process Diagram of the Multicenter Study

© 2023 Yan K et al. *JAMA Network Open.*

eTable 1. Inclusion Criteria for Neonatal Seizure Screening Population

Inclusion Criteria	Description
1. Acute Neonatal Encephalopathy	Newborns showing depressive symptoms due to suspected perinatal asphyxia (chronic or acute), particularly those who underwent cardiopulmonary resuscitation after birth.
2. Cardiac or Pulmonary Risks for Acute Brain Injury and Clinical Encephalopathy	Newborns with significant respiratory conditions such as severe persistent pulmonary hypertension (PPHN), those needing ECMO, and newborns with congenital heart defects requiring early surgery using cardiopulmonary bypass. PPHN and severe congenital heart diseases are both confirmed through bedside echocardiography.
3. CNS Infection	Newborns with laboratory-confirmed meningoencephalitis, or suspected CNS infection, such as clinical evidence in setting of maternal chorioamnionitis, funisitis, group B streptococcus or HSV colonization.
4. CNS Trauma	Newborns admitted with intracranial subarachnoid, subdural, or intraventricular bleeding identified by ultrasound, or suspected CNS injury.
5. Inborn Errors of Metabolism	Newborns with clinical symptoms or family history, suspected or confirmed to have inborn errors of metabolism.
6. Perinatal Stroke	Newborns with clinical symptoms, suspected or confirmed to have perinatal stroke, such as unilateral limb convulsions.
7. Sinovenous Thrombosis	Newborns suspected or confirmed to have sinovenous thrombosis, which is mainly detected by enhanced MRI or CTA in the early stage.
8. Premature Infants	Includes extremely low birth weight infants (weight less than 1000g) or gestational age less than 30 weeks, or preterm infants with other risks of brain damage, such as asphyxia, hypoxia-ischemia, metabolic disorders, infections.
9. Genetic Syndromes	Newborns with clinical symptoms or family history, abnormal features or multiple anomalies, suspected or confirmed to have genetic syndromes.
10. Cerebral Dysgenesis or Malformation	Newborns with cerebral dysgenesis or malformation identified by neuroimaging.

eTable 2. Monitoring Standards of cEEG for Neonates in Neonateal Neurocritical Care Units in China

Key Points	Description
Monitoring Target	The aim is to provide comprehensive, continuous, real-time brain function monitoring to offer necessary medical intervention. The ultimate goal is to achieve brain protection, reduce the incidence of cerebral palsy, and lower the mortality rate.
Spatial Construction	The space should meet the special needs of high-risk newborns, including appropriate temperature and humidity control, as well as noise and light management. Units with the capability should be equipped with ward units or room units. Bed units facilitating brain monitoring operations should also be provided. There should be enough space for medical staff to operate and for family members to accompany.
Monitoring Equipment	In addition to continuous EEG monitors with video recording, it should also include ECG monitors, pulse oximeters, continuous blood glucose monitors, non-invasive ventilators, invasive ventilators, bedside ultrasound, CT, and MRI equipment. This arrangement will provide real-time or regularly updated clinical management data, enabling researchers to timely discover and deal with any potential clinical issues.
Functional Modules	Including ① Standard NICU configuration: ECG monitoring, pulse oximetry, temperature, blood pressure, jaundice, blood sugar, blood gas, trace biochemistry, transcutaneous oxygen and carbon dioxide pressure monitoring devices, resuscitation, non-invasive and invasive mechanical ventilation, and transport of critically ill newborns; ② Essential NNICU equipment: Neurophysiological monitoring (at least 3 sets of video EEG monitors), cerebral oxygen/cerebral blood flow monitor, hypothermia treatment device, and bedside Doppler ultrasound; ③ Supporting equipment for medical institutions: Large-scale neuroimaging diagnostic equipment such as CT and MRI, continuous blood glucose monitors, genetic and molecular diagnostics (second-generation gene sequencing technology, tandem mass spectrometry), peripheral nerve/muscle detection technology (surface electromyography, muscle pathology), neurodevelopmental assessment system, non-invasive cardiac output monitor.
Staffing	The nursing configuration of the monitoring unit should be 1.5 times that of the standard NICU. Team members of the monitoring unit must include several neonatologists, neonatal specialist nurses, a pediatric neurologist, a neuroimaging physician, two neurophysiologists, a genetic counselor, a developmental assessment

© 2023 Yan K et al. *JAMA Network Open.*

	therapist, a pediatric rehabilitation therapist, a clinical nutritionist, and a neurosurgeon. Optional staffing for capable medical institutions can include pediatric otolaryngology and basic neuroscientists.
Training and Assessment	The monitoring unit team, based on the neonatal neurocritical care monitoring training course system of several children's specialty hospitals in North America, should jointly participate in establishing a routine business learning system, completing a cycle of training every year.
Follow-up Management	Capable institutions should consider and improve the accumulation of real-world data in NNICU and the follow-up management system outside the hospital, contributing to the construction of large-scale brain development in newborns.

© 2023 Yan K et al. *JAMA Network Open.*

eTable 3. Diagnostic Criteria for the Etiology of Neonates With Seizures

Etiology	Diagnostic criteria
Intracranial hemorrhage	Intracranial bleeding was detected through neuroimaging examinations (head ultrasound, CT, or MRI) once the vital signs were stable.
Central nervous system infection	Infection was confirmed through cerebrospinal fluid examination, blood culture, and pathogen detection once the vital signs were stable.
Transient metabolic disorder	Abnormal metabolic indicators were detected through blood biochemistry tests, and returned to normal after treatment once the vital signs were stable.
Genetic syndromes	Diagnoses were confirmed through clinical manifestations, family history, and genetic testing.
Ischemic stroke	Ischemic changes were detected through neuroimaging examinations (cranial ultrasound, MRI, or CTA) once the vital signs were stable.
Central nervous system malformation	Structural abnormalities in the brain were detected through neuroimaging examinations (head MRI or CT) once the vital signs were stable.
Inborn errors of metabolism	Diagnoses were confirmed through newborn screening and genetic testing.
Acute Neonatal Encephalopathy (ANE)	After ruling out all the causes above, ANE was confirmed based on neonatal birth history (Apgar score), clinical manifestations (coma, convulsions, difficulty swallowing, respiratory distress, abnormal muscle tone, etc.), postnatal laboratory indicators (umbilical artery blood gas), neuroimaging examinations (edema, softening), and electroencephalograms. If the newborn had a clear perinatal hypoxic-ischemic event, a 5-minute Apgar score of less than 7, or an umbilical arterial blood gas pH of less than 7.0, ANE was given a high consideration.

© 2023 Yan K et al. *JAMA Network Open.*

eTable 4. Seizure Burden in Neonates Under Different PMA and Etiologies

Etiology	PMA	Seizure burden			Total	*P*
		Severe	Moderate	Mild		
	-	426	702	2295	3423	-
Total	Preterm	165	248	786	1199	0.201
	Full-term	261	454	1509	2224	
Acute neonatal encephalopathy	Preterm	67	87	289	443	0.001
	Full-term	95	168	742	1005	
Intracranial hemorrhage	Preterm	40	55	199	294	0.808
	Full-term	42	69	225	336	
CNS infection	Preterm	5	15	70	90	0.323
	Full-term	9	22	173	204	
Transient metabolic	Preterm	2	13	121	136	0.601*
	Full-term	3	8	120	131	
Genetic syndromes	Preterm	15	18	25	58	0.406
	Full-term	34	52	44	130	
Ischemic strokes	Preterm	9	22	26	57	0.885
	Full-term	17	46	61	124	
Unknown	Preterm	8	16	27	51	0.812
	Full-term	20	33	71	124	
CNS malformations	Preterm	7	9	8	24	0.795
	Full-term	26	35	42	103	
Inborn-errors of metabolism	Preterm	12	13	21	46	0.885

© 2023 Yan K et al. *JAMA Network Open.*

Full-term	15	21	31	67

Note: * Fisher's exact test

© 2023 Yan K et al. *JAMA Network Open.*
