# Supplementary material for: Incidence of Neonatal Seizures in China Based on Electroencephalogram Monitoring in Neonatal Neurocritical Care Units
Source: JAMA Netw Open. 2023 Jul 28;6(7):e2326301. doi: 10.1001/jamanetworkopen.2023.26301 (PMC10383014; doi:10.1001/jamanetworkopen.2023.26301)
Supplement: Supplement 2. — Nonauthor Collaborators [file jamanetwopen-e2326301-s002.pdf]

Supplemental Online Content: Nonauthor Collaborators
*First name, last name, and suffix (if applicable) are required and will appear in PubMed.

*Group Name(s): China Neonatal Neuro-Critical Care Network group							
***First Name and Middle Initial(s)**	***Last Name**	***Suffix (eg, Jr, III)**	Academic Degrees	Institution	Location (city, state/province, country)	Role or Contribution, eg, chair, principal investigator	Group (if more than 1 Group listed in the byline) and/or Subgroup (eg, Steering Committee)
Yuan	Shi		MD	Children's Hospital of Chongqing Medical University	Chongqing, China	Site investigators	
Changyi	Yang		MD	Fujian Maternity and Child Health Hospital	Fuzhou, Fujian, China	Site investigators	
Huaping	Zhu		MD	Maternal and Child Health Hospital of Hubei Province	Wuhan, Huzhou, China	Site investigators	
Mingyan	Hei		MD	Beijing Children's Hospital	Beijing, China	Site investigators	
Xing	Feng		MD	Children's Hospital of Soochow University	Suzhou, Jiangsu, China	Site investigators	
Xin	Ding		MD	Children's Hospital of Soochow University	Suzhou, Jiangsu, China	Site investigators	
Zhenlang	Lin		MD	The Second Affiliated Hospital and Yuying Children's Hospital of Wenzhou Medical University	Wenzhou, Zhejiang, China	Site investigators	
Shangqin	Chen		MD	The Second Affiliated Hospital and Yuying Children's Hospital of Wenzhou Medical University	Wenzhou, Zhejiang, China	Site investigators	
Lizhong	Du		MD	The Children's Hospital, Zhejiang University School of Medicine	Hangzhou, Zhejiang, China	Site investigators	
