# Supplementary material for: Incidence of Neonatal Seizures in China Based on Electroencephalogram Monitoring in Neonatal Neurocritical Care Units
Source: JAMA Netw Open. 2023 Jul 28;6(7):e2326301. doi: 10.1001/jamanetworkopen.2023.26301 (PMC10383014; doi:10.1001/jamanetworkopen.2023.26301)
Supplement: Supplement 3. — Data Sharing Statement [file jamanetwopen-e2326301-s003.pdf]

Data Sharing Statement

Yan. Incidence of Neonatal Seizures in China Based on Electroencephalogram Monitoring in Neonatal Neurocritical Care Units. *JAMA Netw Open*. Published July 28, 2023. doi:10.1001/jamanetworkopen.2023.26301

Data
Data available: Yes
Data types: Deidentified participant data
How to access data: Wenhao Zhou has full access.(zhouwenhao@fudan.edu.cn)
When available: With publication

Supporting Documents
Document types: None

Additional Information
Who can access the data: For researcher
Types of analyses: For scientific reseach
Mechanisms of data availability: with a signed data access agreement
